# Evaluating ChatGPT's recommendations for systematic treatment decisions in recurrent or metastatic head and neck squamous cell carcinoma: Perspectives from experts and junior doctors

**DOI:** 10.1002/ijc.70001

**Published:** 2025-07-19

**Authors:** Danfang Yan, Lihong Wang, Liming Huang, Kejia Cheng, Yu Huang, Yangyang Bao, Xin Yin, Mengye He, Huiyong Zhu, Senxiang Yan

**Affiliations:** ^1^ Department of Radiation Oncology, the First Affiliated Hospital, College of Medicine Zhejiang University Zhejiang Hangzhou China; ^2^ Department of Oncology, The Affiliated People's Hospital Fujian University of Traditional Chinese Medicine Fuzhou Fujian China; ^3^ Department of Otolaryngology, the First Affiliated Hospital College of Medicine, Zhejiang University Zhejiang Hangzhou China; ^4^ Department of Oncology, Tongji Hospital, Tongji Medical College Huazhong University of Science and Technology Wuhan China; ^5^ Department of Oncology the First Affiliated Hospital, College of Medicine, Zhejiang University Zhejiang Hangzhou China; ^6^ Department of Oral and Maxillofacial Surgery, the First Affiliated Hospital, College of Medicine Zhejiang University Zhejiang Hangzhou China

**Keywords:** artificial intelligence, ChatGPT, head and neck squamous cell carcinoma, multidisciplinary teams

## Abstract

This study evaluates ChatGPT‐4's potential as a decision‐support tool in the treatment of recurrent or metastatic head and neck squamous cell carcinoma (HNSCC). The study involved 12 retrospectively chosen patients with detailed clinical, tumor, treatment history, imaging, pathology, and symptomatic data. ChatGPT‐4, along with six experts and 10 junior oncologists, assessed these cases. The AI model applied the 8th edition AJCC TNM criteria for tumor staging and proposed treatment strategies. Performance was quantitatively rated on a 0–100 scale by both expert and junior oncologists, with further analysis through statistical scoring and intraclass correlation coefficients. Findings revealed that ChatGPT‐4 achieved an 83.3% accuracy rate in tumor staging with two instances of mis‐staging. Junior doctors rated its staging performance highly, showing strong consensus on language capabilities and moderate on learning assistance. Experts rated ChatGPT‐4's treatment strategy: high agreement on subject knowledge (median 86, mean 84.7), logical reasoning (median 83, mean 82), and analytical skills (median 85, mean 82); moderate on ChatGPT‐4's usefulness for treatment decision (median 80, mean 77) and its recommendations (median 80, mean 76.8). Junior doctors rated ChatGPT‐4 higher in treatment strategy (medians above 85) with limited consensus (subject knowledge: median 88, mean 84.5; logical reasoning: median 90, mean 83.2; analytical skills: median 90, mean 82.5; usefulness: median 85, mean 81.8; agreements for: median 85, mean 80.4). ChatGPT is proficient in tumor staging but moderately effective in treatment recommendations. Nonetheless, it shows promise as a supportive tool for clinicians, particularly for those with less experience, in making informed treatment decisions.

## Graphical abstract

What's new?

While artificial intelligence (AI) can potentially support clinical decision‐making in recurrent head and neck squamous cell carcinoma (HNSCC), its reliability and accuracy require extensive investigation. Here, the performance of ChatGPT‐4 as a decision‐support tool for staging and treatment planning in HNSCC was assessed. The tool was 83% accurate in tumor staging. Experts rated the tool highly on subject knowledge, reasoning, and analytical ability. Treatment decisions, however, received only moderately favorable ratings. Junior doctors rated the tool highly, though perceptions varied considerably. The findings suggest that ChatGPT‐4 is proficient in tumor staging and is a promising support tool for HNSCC.

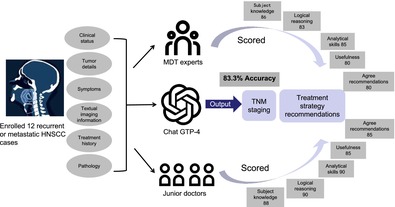

**Abbreviations**
AI: Artificial intelligence
CPS: Combined positive score
HNSCC: Head and neck squamous cell carcinoma
HPV: Human papillomavirus
ICC: Intraclass correlation coefficient
IQR: Interquartile range
MDTs: Multidisciplinary teams
OPC: Oropharyngeal cancer
PD‐L1: PD‐1 ligand 1
TNM: Tumor, Node, Metastasis

## 1 INTRODUCTION

Artificial intelligence (AI) has been widely applied in the medical field, particularly in medical auxiliary diagnosis.^1^, ^2^, ^3^ With learning from past cases, AI has developed discriminant models for various diseases, aiding in faster diagnosis, reducing the workload of medical professionals, and enhancing efficiency. The emergence of AI technologies like ChatGPT has brought significant advancements and challenges.^4^ ChatGPT, powered by unsupervised learning, generates human‐like text and engages in informative dialogues.^5^, ^6^ It has shown potential in medical education, even approaching the passing level for the United States Medical Licensing Examination.^7^ Furthermore, it has revolutionized medical writing, data gathering, literature searches, and preliminary document generation.^8^ Despite its potential benefits in the medical sector, doubts persist due to ChatGPT's lack of critical thinking and occasional provision of redundant or arbitrary information.^9^, ^10^

Head and neck squamous cell carcinoma (HNSCC) encompasses various malignancies, including those affecting the paranasal sinuses, larynx, hypopharynx, oropharynx, and oral cavity. Treatment of locoregional HNSCCs typically involves radical surgery, radiotherapy, or a combination therapy. However, recurrence or metastasis is common, necessitating complex management strategies.^11^, ^12^ In recurrent or metastatic HNSCC (R/M HNSCC) cases that are not amenable to local therapies like surgery or radiotherapy, the standard first‐line treatment options include the EXTREME regimen (comprising cetuximab, platinum, and 5‐fluorouracil)^13^ or the KEYNOT‐048 regimen (involving mono‐pembrolizumab immunotherapy or pembrolizumab in combination with chemotherapy).^14^ PD‐1 ligand 1 (PD‐L1) expression on tumor cells or associated immune cells has been associated with better outcomes for pembrolizumab.^15^ The KEYNOTE‐048 study has validated that pembrolizumab in combination with platinum and 5‐Fu is an effective first‐line treatment for recurrent or metastatic HNSCC in populations with a PD‐L1 combined positive score (CPS) of 20 or more, CPS of 1 or more, and in the total population. This is especially relevant for patients having low PD‐L1 expression or only recurrent disease. In contrast, for PD‐L1‐positive cancers associated with a lower symptom burden, pembrolizumab monotherapy may be the preferred option.^14^ However, the 2021 TPEXTREME study affirmed the efficacy of cetuximab, platinum, and taxanes (TPEx regimen) as another viable first‐line option for R/M HNSCC, yielding similar therapeutic outcomes to those of the KEYNOTE‐048 regimen.^16^

Considering the current progress in clinical research regarding targeted and immunotherapies for R/M HNSCC, optimizing the overall management strategy to maximize patient survival benefits requires careful consideration of treatment sequencing. Multidisciplinary teams (MDTs) play a crucial role in oncologic disease management,^17^ particularly in the complex landscape of HNSCC treatment.^18^ Decisions for R/M HNSCC staging and treatment hinge on factors like treatment history, disease stage, tumor location, and patient status.^19^, ^20^

Given the potential of big language models in clinical decision‐making and medical education, we explored ChatGPT's role in decision‐making associated with R/M HNSCC staging and treatment. We proposed the hypothesis that ChatGPT could perform on par with MDT experts in providing treatment strategies that align with current guidelines, aiming to maximize patient survival.

## 2 MATERIALS AND METHODS

### 2.1 Selection of patients

In this retrospective study, we conducted a random selection of 12 cases of R/M HNSCC who were treated in our institution during a time period spanning from January 2023 to July 2023. The inclusion criteria encompassed (1) the presence of R/M HNSCC disease; (2) prior history of radical surgery or radiotherapy for the initial onset; (3) a recommendation from the MDT specializing in head and neck cancer; and (4) the availability of the patient's informed consent.

### 2.2 Dialogue with ChatGPT

We retrospectively reviewed electronic patient records and provided summaries of 12 cases to ChatGPT version 4.0 (ChatCPT‐4, February 2023). To ensure consistency, completeness, and minimize bias in the input data, we used a standardized template for each of the 12 patients, carefully filling in all relevant information as required. Each case was presented individually in a separate chat session, succinctly detailing information such as age, gender, symptoms at diagnosis, Karnofsky Performance Status, previous treatment history (including whether the patient underwent surgery, radiotherapy, or chemotherapy, along with specifics of the treatment regimen), textual imaging results, PD‐L1 expression status, and the duration between recurrence or metastasis and initial onset. Moreover, both the input and output information were independently double‐checked by two doctors to prevent any data point loss.

Patient identification details were withheld from ChatGPT‐4. Two precise inquiries were directed to the system: (1) “What is the clinical staging for this patient?” and (2) “What is the best treatment strategy for this patient?” ChatGPT‐4's responses were documented, and identical case data and complete chat records were disseminated to both experts and junior doctors for evaluation.

### 2.3 Selection of experts and junior doctors

Our MDT specializing in head and neck cancers comprises surgeons, medical oncologists, radiation oncologists, radiologists, pathologists, and head and neck tumor rehabilitation specialists, each possessing over 10 years of clinical experience. We regard our institutional MDT as a benchmark for evidenced‐based decisions and achieving consensus through multidisciplinary collaboration. Each R/M HNSCC patient admitted to our institution undergoes evaluation by this MDT. For this study, there were four experts from our MDT (two surgeons, one radiation oncologist, and one medical oncologist) and two external independent senior experts from another domestic tertiary hospital (one radiation oncologist and one medical oncologist), all of whom evaluated ChatGPT‐4's responses in comparison to their formal decisions.

Standardized residency training for oncologists is an essential aspect for improving the ability of cancer treatment. Ten junior doctors were recruited from the cohort of newly appointed standardized training oncology physicians at the First Affiliated Hospital of the College of Medicine, Zhejiang University. This group comprised four junior doctors with 3 years of clinical experience, three with 2 years, and three with 1 year of practice.

### 2.4 Evaluation process

Experts compared ChatGPT‐4's responses to the treatment decisions made by the MDT for each of the 12 cases, using the MDT decisions as the benchmark. Ratings ranged from 0 to 100, with ‘0–20’ indicating complete disagreement, ‘21–40’ reflecting disagreement, ‘41–60’ indicating neutrality, ‘61–80’ showing moderate agreement, and ‘81–100’ representing high agreement. Evaluation criteria included how well the patient's functional status and treatment history were factored into the decision‐making, the depth of subject knowledge demonstrated, and the logical coherence of responses.

**FIGURE 1:**
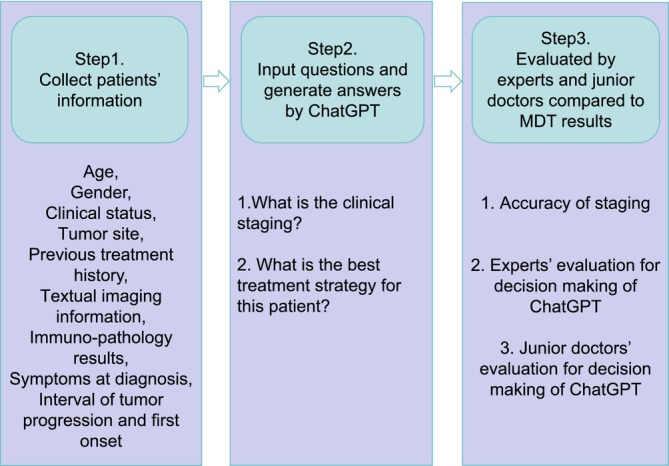
Summary of study workflow.

Similarly, junior doctors were tasked with evaluating ChatGPT‐4's performance through two questionnaires, one for assessing tumor staging and the other for assessing systematic therapy recommendations. The rating system aligned with that used by the experts. Additionally, junior doctors assessed language proficiency and the educational value of ChatGPT‐4's responses. Treatment recommendations were evaluated based on tumor staging, proposed treatment plans, and the functional status of the patient. The study workflow is illustrated in Figure 1. Finally, the agreement between experts and junior doctors was analyzed separately. Every one (either expert or junior doctor) scored the same questionnaires twice at different times (the interval time exceeded 1 month), the final score results were the average of the two scores, and the intra‐observer consistency were calculated.

### 2.5 Statistics

We conducted a statistical analysis using SPSS 26.0. Ordinal variables were summarized as the mean and median with the interquartile range (IQR), and a comparison using the Mann–Whitney *U* test was performed when applicable. The agreement between experts and junior doctors was assessed using the intraclass correlation coefficient (ICC). Both the intra‐observer and inter‐observer's ICC were calculated. ICC values below 0.5 were considered poor, between 0.5 and 0.75 moderate, between 0.75 and 0.9 good, and above 0.9 excellent. The statistical significance was determined by a two‐sided *p*‐value <0.05 for hypothesis testing.

## 3 RESULTS

### 3.1 Patients

Among the 12 participants, comprising 10 males and two females, with a median age of 59 years (ranging from 46 to 76 years) who were involved in this research. The tumor locations included four oral cavity, three oropharynx, two hypopharynx, two maxillary sinus, and one larynx. Seven were identified as having recurrent cancer, three had both recurrent and metastatic cancer, and two were diagnosed solely with metastatic disease. The median duration between tumor progression and initial onset was 12 months (ranging from 3 to 36 months) (refer to Data S1).

### 3.2 ChatGPT‐4's role in tumor staging analysis

Utilizing the 8th edition of the Tumor, Node, Metastasis (TNM) Classification of Malignant Tumors system from the American Joint Committee on Cancer (AJCC), staging of HNSCC was conducted. ChatGPT‐4 addressed the initial inquiry regarding clinical staging post‐recurrence or metastasis by providing a TNM diagnosis and clinical staging based on the AJCC 8th edition guidelines for head and neck cancer. The process involved outlining the definitions of T (tumor), N (nodes), and M (metastasis) classifications and subsequently delving into the specific case descriptions provided in the image text. Through a detailed analysis, ChatGPT‐4 diagnosed and explained the specific T, N, and M stages. For instance, it identified a tumor's size and invasion of the lingual surface of the epiglottis as characteristics of T3, bilateral cervical lymph node metastasis without extracapsular spread as N2c, and lung metastasis as M1. These findings were synthesized into a comprehensive TNM classification and clinical staging diagnosis, as exemplified by the designation of T4aN2cM0 for recurrent laryngeal cancer, corresponding to Stage IVA for laryngeal cancer.

In the descriptions of T and N staging, ChatGPT‐4 highlighted the importance of providing key information, such as the tumor size and involvement of surrounding tissues, to accurately define T3, T4a, or T4b stages. Furthermore, it emphasized the necessity for specific details regarding metastatic lymph nodes, including their location (unilateral or bilateral, ipsilateral, or contralateral) and characteristics such as diameter and extracapsular invasion. For oropharyngeal cancer (OPC), ChatGPT‐4 noted the significance of determining the expression status of the p16 protein to differentiate between human papillomavirus (HPV)‐positive and HPV‐negative OPC cases.

### 3.3 ChatGPT‐4's role in developing a treatment strategy

The provided paragraphs discuss the approach taken by ChatGPT‐4 in addressing treatment strategies for R/M HNSCC. ChatGPT‐4 offered staging diagnoses, treatment plan recommendations, and considerations based on the functional status for 12 cases. ChatGPT‐4 systematically analyzed each case, categorizing information into sections such as past medical history, current clinical status, symptoms, and imaging results. Subsequently, it outlined potential treatment options, including re‐evaluation for surgery, systemic therapy, symptomatic management, clinical trials, and multidisciplinary evaluation.

However, instead of prescribing specific treatment options, ChatGPT‐4 emphasized the importance of engaging in further MDT discussions to determine the most suitable treatment regimen. It stressed the significance of patient preferences and informed decision‐making regarding the potential benefits, risks, and quality of life implications associated with each treatment option.

In terms of systemic treatment regimens, ChatGPT‐4 provided a comprehensive list of drugs for HNSCC, considering factors such as PD‐L1 expression levels. It recommended immunotherapy with pembrolizumab (Keytruda) and nivolumab (Opdivo) for cases with a PD‐L1 CPS >1. In situations where immunotherapy was not viable, platinum‐based therapy and taxanes like paclitaxel or docetaxel were suggested. Additionally, targeted therapy with cetuximab, either alone or combined with chemotherapy, was proposed.

For patients experiencing symptoms, ChatGPT‐4 advocated for a multimodal approach to supportive and palliative care, encompassing pain management, nutritional support, and possibly palliative radiation based on the recurrence's location and previous treatments.

In conclusion, ChatGPT‐4 stressed the importance of considering factors such as tolerability, potential side effects, and efficacy when selecting systemic treatments. It underscored the necessity of thorough discussions with treating oncologists and MDTs to tailor treatment strategies to each patient's unique circumstances and preferences.

### 3.4 Evaluation of ChatGPT‐4 by experts and junior doctors

#### 3.4.1 Experts' evaluation for ChatGPT‐4's TNM staging performance

Experts, as members of MDT, focused their evaluation on the accuracy of ChatGPT‐4's TNM staging performance within the context of a MDT setting. Among the 12 patients assessed, ChatGPT‐4 demonstrated an overall staging accuracy of 83.3%. Specifically, inaccuracies were noted in one T staging and one N staging, resulting in an overall accuracy of 91.7% for combined T and N staging. Notably, ChatGPT‐4 achieved 100% accuracy for M staging and clinical staging.

An incorrect T‐staging occurred in a patient with a maxillary sinus tumor, attributed to an error in the descriptive text regarding T4b. The description for T4b staging provided by ChatGPT‐4 included local invasion of structures, such as the orbital apex, dura, brain, nasopharynx, clivus, or central compartment structures. The misclassification stemmed from erroneously including the cribriform plate among structures indicative of T4b invasion. According to the 8th edition of AJCC TNM staging, such invasion should classify as T4a instead of T4b.

Similarly, an incorrect N‐staging was observed in a patient with recurrent oral cancer, where ChatGPT‐4 staged N3b as N3a. This misclassification resulted from an oversight regarding the definition of N3a and N3b in the context of oral cancer, particularly concerning extracapsular spread.

Despite these discrepancies, experts did not extensively evaluate other aspects of ChatGPT‐4's TNM staging performance, instead focusing primarily on the accuracy of staging classifications.

#### 3.4.2 Junior doctors' evaluation for ChatGPT‐4's TNM staging performance

The junior doctors found TNM staging for HNSCC to be particularly challenging due to its complexity. Each subsite has unique staging criteria based on anatomical variations and tumor extent. For instance, OPC staging differs depending on whether HPV is present. Consequently, junior doctors struggle to grasp and memorize the intricacies of HNSCC staging.

Junior doctors evaluated ChatGPT‐4's staging performance considering both its language capabilities and utility in learning. The former aspect assessed ChatGPT‐4's command of subject knowledge, logical reasoning, inductive reasoning and analytical skills. The latter aspect examined five factors: ChatGPT‐4's practicability, effectiveness in helping users understand and reinforce important concepts, role in enhancing independent staging abilities, contribution to active learning, and impact on enthusiasm and interest in learning.

**FIGURE 2:**
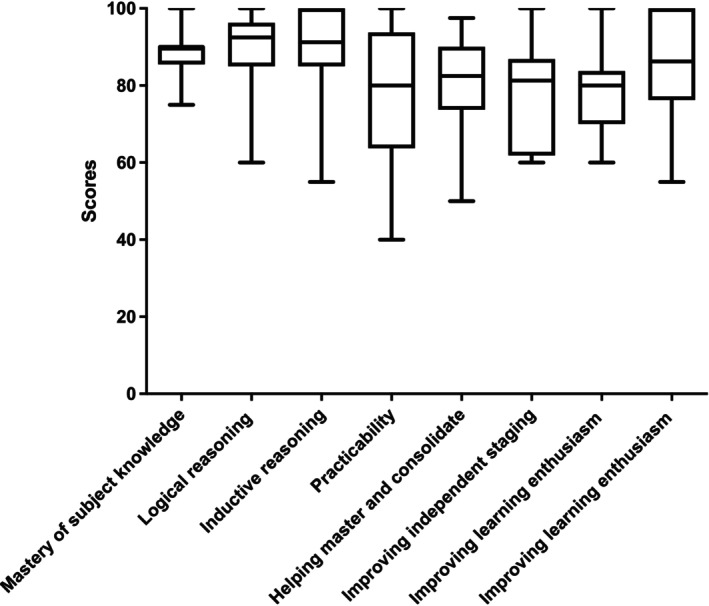
ChatGPT‐4's TNM staging evaluation scores provided by junior doctors.

The evaluation scores, including median, mean, IQR, and standard deviation, are presented descriptively in Table 1 and Figure 2. Overall, junior doctors rated ChatGPT‐4's language abilities highly, with median and mean scores surpassing 85, indicating strong consensus. However, due to occasional inaccuracies in staging, junior doctors' ratings regarding ChatGPT‐4's effectiveness in facilitating learning were slightly lower. The median score indicating ChatGPT‐4's contribution to learning assistance was 80, suggesting moderately agreement. The intra‐observer's ICC exceeded 90.

**TABLE 1:** Junior doctors' evaluation for TNM staging.

Questionnaire items	Median	Mean	SD	IQR	Intra‐observer's ICC
Evaluation of ChatGPT‐4					
Mastery of subject knowledge	90	87.9	7.5	85.5–90	0.92
Logical reasoning	92	88.8	12.0	85–96	0.97
Inductive reasoning and analytical skills	90	88	14.5	85–100	0.98
How ChatGPT‐4 assists in learning					
Practicability	80	75.8	20.2	63.8–93.8	0.98
Helping master and consolidate the important and difficult points	80	80	13.9	73.8–90	0.98
Improving the ability of independent staging	82	77.5	13.9	62–87	0.97
Promoting active learning	80	78	11.3	70–83.8	0.98
Improving learning enthusiasm and interest	86	85	15.6	76.3–100	0.97

Abbreviations: ICC, intraclass correlation coefficient; IQR, intra‐quartile range.

#### 3.4.3 Experts' evaluation of ChatGPT‐4's treatment strategy performance

The experts' evaluation of ChatGPT‐4's treatment strategy encompassed its mastery of subject knowledge, logical thinking ability, and inductive reasoning and analytical skills. Furthermore, experts assessed whether ChatGPT‐4's recommendations were useful for making treatment decisions and their level of agreement with the provided advice. Evaluation scores were presented descriptively, including median, mean, IQR, and standard deviation. Those scores were subjected to comparative analysis using Mann–Whitney *U* or Kruskal–Wallis testing (refer to Table 3). Each expert evaluated the same questionnaires twice, the median of intra‐observer's ICC for experts was 0.95 (with a range of 0.80–0.99).

**FIGURE 3:**
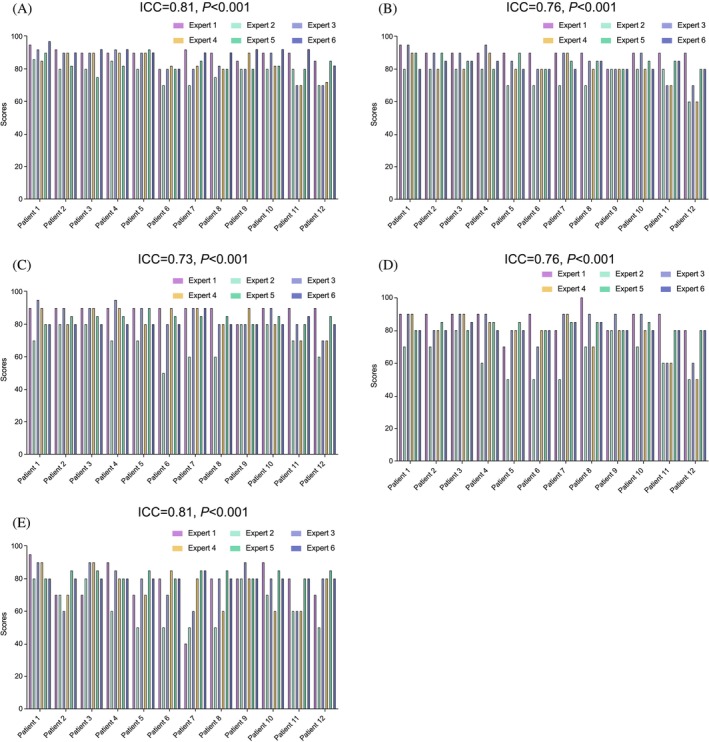
Bar plots representing the ratings per patient and per expert.

Table 2 and Figure 3 illustrate the inter‐rater agreement for each evaluated aspect. In terms of ChatGPT‐4's mastery of subject knowledge, logical thinking ability, and analytical capabilities, experts rated the output as highly agreement, with median scores of 86 (IQR 81.2–88.2), 83 (IQR 79.6–86.8), and 85 (IQR 74.4–85.8), respectively. Agreement among the experts was good to moderate, with ICC values of 0.81, 0.76, and 0.73, respectively.

**TABLE 2:** ChatGPT‐4 evaluation scores provided by six experts.

Questionnaire items	Median	Mean	SD	IQR	ICC
Mastery of subject knowledge	86	84.7	5.7	81.2–88.2	0.81
Logical thinking ability	83	82.6	5.5	79.6–86.8	0.76
Inductive reasoning and analytical skills	85	82	5.4	74.4–85.8	0.73
Useful for treatment decisions	80	77	8.9	71.5–84.0	0.76
Agree with ChatGPT‐4's treatment advice	80	76.8	9.3	71.4–83.8	0.81

Abbreviations: ICC, intraclass correlation coefficient; IQR, intra‐quartile range.

Regarding the utility of ChatGPT‐4's recommendations for treatment decisions, experts deemed them to be moderately agreement, with a median score of 80 (IQR 71.5–84.0), and exhibited good agreement (ICC 0.69). Similarly, for overall agreement with ChatGPT‐4's treatment advice, experts rated the recommendations as moderately agreement, with a median score of 80 (IQR 71.4–83.5), and demonstrated good agreement (ICC 0.81).

#### 3.4.4 Junior doctors' evaluation of ChatGPT's treatment strategy performance

Ten junior doctors were involved in a questionnaire aimed at assessing ChatGPT‐4's treatment strategy. The evaluation encompassed five primary aspects: ChatGPT‐4's grasp of subject knowledge, its capacity for logical thinking, inductive reasoning, and analytical skills, its suitability for study purposes, and the agreement level with the treatment advice provided by ChatGPT‐4.

While the questionnaire shared similarities (only slight differences in content) with the one administered to the experts, it was tailored to better suit the characteristics and preferences of the junior doctors. This customization ensured that the questionnaire content resonated more closely with the clinical experiences and contexts of the junior doctors.

The differences in content between the questionnaire for the junior doctors and that for the experts stemmed from the distinct perspectives, priorities, and concerns of these two groups. For instance, the junior doctors' questionnaire might have included inquiries addressing their specific learning needs, areas of interest, or challenges encountered during their clinical practice, which may not have been as pertinent for the experts.

Overall, the survey questionnaire was thoughtfully designed to prioritize the questions most relevant to the respondents' concerns and experiences, whether they were junior doctors or experts. Every junior doctor scored the same questionnaires twice, and the median of intra‐observer's ICC for junior doctors was 0.96 (with a range of 0.75–0.99).

Table 3 presents the score results and inter‐rater agreement for each evaluated aspect. Across all items, ChatGPT‐4's output received favorable evaluations, with median scores ranging from 85 to 90. Specifically, the median scores for mastery of subject knowledge, logical thinking ability, and inductive reasoning and analytical skills were 88 (IQR 80.1–89.5), 90 (IQR 80.6–87.8), and 90 (IQR 78.6–96.5), respectively. Additionally, its usefulness for study purposes and agreement with treatment advice received median scores of 85 (IQR 76.5–86.9) and 85 (IQR 75.7–86.2), respectively.

**TABLE 3:** ChatGPT‐4 evaluation scores provided by 10 junior doctors.

Questionnaire items	Median	Mean	SD	IQR	ICC
Mastery of subject knowledge	88	84.5	5.6	80.1–89.5	0.64
Logical thinking ability	90	83.2	7.1	80.5–87.8	0.22
Inductive reasoning and analytical skills	90	82.5	7.9	78.6–96.5	0.52
Useful for study	85	81.8	6.8	76.5–86.9	0.32
Satisfied with ChatGPT‐4's treatment advice	85	80.4	7.5	75.7–86.2	0.30

Abbreviations: ICC, intraclass correlation coefficient; IQR, intra‐quartile range.

However, despite the positive evaluations, the agreement among junior doctors was poor, as indicated by the relatively low ICC values ranging from 0.22 to 0.64. This suggests some variability in perceptions among junior doctors regarding ChatGPT‐4's treatment strategy.

## 4 DISCUSSION

The study evaluated ChatGPT‐4's performance in providing TNM staging diagnoses and treatment recommendations for R/M HNSCC. While ChatGPT‐4 demonstrated accuracy in clinical staging, it exhibited limitations in precisely subdividing T and N staging. Moreover, its treatment strategy and regimen recommendations were deemed the well‐agreement by junior doctors but only moderately agreement by experts.

Multidisciplinary discussions among oncology teams are crucial for optimizing treatment outcomes in cancer patients and standardizing treatment protocols.^21^ However, the complexity of HNSCC staging, with variations across subsites based on anatomical differences and tumor extent, poses challenges.^22^ Notably, oropharyngeal tumors require additional assessment for HPV status in examining p16 expression by immunohistochemistry, adding further complexity to staging protocols.^23^, ^24^, ^25^ Pre‐treatment MDT discussions help unify staging approaches and enable consensus building among diverse medical specialties regarding treatment plans.

Nevertheless, logistical constraints such as time, resource availability, economic costs, and geographic disparities limit the feasibility of MDT organization.^19^, ^20^ In this context, AI‐based tools like ChatGPT‐4, which leverage deep learning algorithms, could serve as valuable adjuncts or aids, particularly for tumor centers lacking specialized expertise or resources. ChatGPT‐4 has the potential to enhance clinical decision‐making abilities by providing guidance to healthcare providers. This is especially beneficial for junior doctors who may have limited clinical experience and incomplete familiarity with treatment guidelines. ChatGPT‐4 can help bridge these knowledge gaps and promote standardized operating procedures within medical practice.

The accuracy of ChatGPT‐4 for determining clinical staging and M staging was found to be 100%, indicating a high level of precision in these areas. However, when it came to T and N staging, ChatGPT‐4 did not achieve complete precision. Specifically, there were two instances where errors occurred: In one case, ChatGPT‐4 incorrectly classified a tumor as T4a instead of T4b, and in another case, it provided an incorrect stage description, labeling N3b instead of N3a. These inaccuracies could potentially mislead junior doctors, especially if they rely too heavily on ChatGPT‐4 for staging information. Given that ChatGPT does not reach 100% accuracy in the intricate details of tumor staging, clinicians should exercise caution and not depend solely on its outcomes. These inaccuracies underscore the necessity for additional refinement of AI models to boost their precision in complex clinical contexts. Looking ahead, enhancing ChatGPT's capabilities by integrating more comprehensive, multimodal data, such as imaging and pathology, could potentially improve its performance in tumor staging.

Despite these errors, junior doctors expressed a high level of agreement with ChatGPT‐4 overall, particularly in terms of its mastery of subject knowledge, logical thinking ability, and inductive reasoning and analytical skills. The median scores for these aspects were all 90, indicating strong agreement with both the staging and its interpretation. However, when evaluating how ChatGPT‐4 aids in learning, including its practicability, effectiveness in mastering and consolidating important points, enhancing independent staging abilities, promoting active learning, and increasing enthusiasm and interest in learning, the median scores were lower, at 80 points. This discrepancy suggests that while junior doctors appreciate ChatGPT‐4's knowledge and abilities, they are less satisfied with its impact on their learning process, likely due to the presence of staging errors that could lead to confusion and hinder learning progress.

When it comes to advising on treatment strategies, ChatGPT‐4 demonstrates a commendable level of clarity and depth of knowledge, which proves beneficial for junior doctors seeking guidance in their studies and decision‐making processes. Its recommendations and analyses of treatment strategies are thorough and comprehensive, which is particularly advantageous for patients with metastatic lesions or those necessitating systemic treatment.

However, ChatGPT‐4's proficiency in recommending local treatments for patients experiencing local or regional recurrence falls short. Additionally, there appears to be a lack of specificity in tailored treatment strategies, indicating a need for a more nuanced analysis that incorporates previous treatment history and additional patient data. Furthermore, the recommendations overlook crucial factors such as the patient's overall physical condition, past treatment experiences, tumor site, and staging. For example, the same local treatment suggestions are offered for both T3 and T4 lesions in patients experiencing local recurrence. In essence, while the recommendations for systemic therapy are comprehensive, there's a noticeable absence of specific guidance on local treatments, like palliative radiotherapy for symptom management. This discrepancy may be attributed to the current data, which emphasizes systemic therapies more heavily.^13^, ^14^, ^16^ It also arises from limitations in training data, algorithm constraints, and input formatting. A key distinction between ChatGPT and multidisciplinary teams (MDTs) is evident here. MDTs consist of seasoned experts across various specialties who devise personalized treatment plans tailored to clinical guidelines, clinical research, personal experience, each patient's specific health condition, and economic factors. In contrast, ChatGPT primarily bases its treatment recommendations on existing guidelines, clinical research, and available evidence. For local treatment strategies with scant clinical research, its recommendations are less robust, and it may not adequately consider individual patient factors—a crucial aspect for clinicians to be mindful of when employing AI for treatment advice. Additionally, ChatGPT lacks the capability to offer specific and tailored treatment recommendations that account for patient‐specific factors like comorbidities, personal preferences, and performance status, although it does acknowledge the importance of clinicians making personalized treatment decisions based on each patient's unique circumstances.

In the evaluation conducted by experts on treatment strategies, ChatGPT‐4 performed well in three key parameters: mastery of subject knowledge, logical thinking ability, and inductive reasoning and analytical skills, garnering moderate agreement among the six experts from various disciplines and centers. However, when assessing whether ChatGPT‐4 was useful for treatment decisions and agreement with its treatment advice, experts rated it moderately, with median and mean scores of 80 and 77, and 76 and 74, respectively. These results underscored certain limitations in ChatGPT‐4's treatment suggestions, reaffirming that ChatGPT‐4 cannot fully replace the outcomes of MDT discussions.^26^

Conversely, junior doctors evaluated treatment strategies as excellent, with median and mean scores for all five parameters exceeding 81. Nevertheless, there was poor agreement among junior doctors, with the ICCs for all five items falling below 0.25. The low ICC among junior doctors indicates variability in their evaluations, potentially due to differences in expertise, clinical experience, or familiarity with HNSCC treatment guidelines. This variability might be because some junior doctors, due to their limited experience, might lean more on ChatGPT's outputs, whereas others may evaluate its recommendations more critically based on their existing knowledge. This divergence in professional knowledge and understanding of treatment decisions has resulted in notable discrepancies in scoring, in stark contrast to the more uniform evaluations observed among experts.

Despite these challenges, our findings underscored ChatGPT‐4's potential role in facilitating clinical decision‐making and aiding junior doctors in rapidly acquiring subject knowledge. Indeed, chatbots such as ChatGPT‐4 have the capability to swiftly provide relevant information across various aspects of healthcare, including diagnosis, treatment options, patient medical history, and potential side effects. Leveraging natural language processing and large datasets, these chatbots can effectively analyze and interpret complex medical information to offer concise and accurate responses to user inquiries. For diagnosis, chatbots can help users understand symptoms and potential conditions based on the provided information, serving as an initial point of reference for further investigation by healthcare professionals. Additionally, they can offer insights into the various treatment options that are available for specific conditions, including medications, therapies, and procedures, enabling users to make informed decisions about their healthcare.

However, it is imperative for ChatGPT‐4 to maintain timely updated information to avoid adverse impacts on clinical treatment outcomes. Therefore, additional interaction and extended discourse with chatbots could potentially enhance their performance. In future studies, employing alternative questioning methods that align with the information processing capabilities of chatbots could lead to more accurate results.^27^, ^28^ To minimize bias and enhance performance, it is crucial to provide ChatGPT with standardized, comprehensive, and consistent information. Nevertheless, limitations in AI processing, such as interpreting context and variability in decision‐making, may still affect its performance. Future research could investigate various data presentation methods and compare ChatGPT's responses across different levels of case complexity to better assess and enhance its decision‐making skills. Additionally, while ChatGPT‐4 is trained on a wide array of data sources, including publicly accessible clinical guidelines and literatures, it may not fully account for variations in clinical guidelines that differ by region or institution. Since clinical practices, particularly in cancer treatment, can vary based on local protocols, institutional preferences, or regional guidelines, recognizing these potential discrepancies is crucial for understanding how they might influence the model's decision‐making process. The ongoing advancement of AI continually pushes the boundaries of what healthcare professionals envision, yet it remains clear that it cannot supplant the expertise and technology wielded by clinical doctors.^29^

This study has several limitations. First, it only compares ChatGPT‐4 with the traditional MDT model, without evaluating other AI models. This gap will be addressed in our future research. Second, the sample size of the study is not sufficiently large; however, it is intended as an initial proof‐of‐concept investigation to assess the viability of using ChatGPT in comparison to the traditional MDT model, drawing on feedback from various experts and junior doctors.

## 5 CONCLUSIONS

In conclusion, this study assessed ChatGPT‐4's effectiveness in staging and treatment recommendations for R/M HNSCC, as evaluated by both experts and junior doctors. While ChatGPT‐4 demonstrated proficiency in tumor staging, its performance in treatment recommendations was deemed moderate. It lacks the precision and personalization required to substitute expert opinions. Nonetheless, ChatGPT‐4 can serve as a valuable supplementary tool for clinicians, particularly for less‐experienced doctors, thereby aiding in the treatment decision‐making process. Despite limitations in its clinical application, ChatGPT represents a future trend in medicine and is poised to bring about transformative changes in the field.

## AUTHOR CONTRIBUTIONS

**Danfang Yan:** Writing – original draft; conceptualization; writing – review and editing; investigation; funding acquisition; formal analysis; data curation; resources. **Lihong Wang:** Methodology; writing – original draft; software; investigation; writing – review and editing; data curation. **Liming Huang:** Data curation; investigation; visualization. **Kejia Cheng:** Investigation; visualization. **Yu Huang:** Investigation. **Yangyang Bao:** Investigation; visualization. **Xin Yin:** Investigation. **Mengye He:** Investigation. **Huiyong Zhu:** Investigation; conceptualization; writing – review and editing; project administration; validation; resources. **Senxiang Yan:** Conceptualization; investigation; writing – review and editing; project administration; supervision; resources.

## FUNDING INFORMATION

This study is supported by the China National Science Foundation (Grant numbers 62307031).

## CONFLICT OF INTEREST STATEMENT

All other authors declare no competing interests.

## ETHICS STATEMENT

This study was approved by the Ethics Committee of the First Affiliated Hospital of College of Medicine at Zhejiang University (Reference number: IIT20240163B‐R1).

## Supporting information


**DATA S1.** Supporting information.

## Data Availability

Research data are stored in an institutional repository and will be shared upon reasonable request to the corresponding author.
