# Supplementary material for: Evaluating ChatGPT's recommendations for systematic treatment decisions in recurrent or metastatic head and neck squamous cell carcinoma: Perspectives from experts and junior doctors
Source: Int J Cancer. 2025 Jul 19;157(9):1888–97. doi: 10.1002/ijc.70001 (PMC12407031; doi:10.1002/ijc.70001)
Supplement: Supplementary file 1 — DATA S1. Supporting information. [file IJC-157-1888-s001.pdf]

Evaluating ChatGPT's Recommendations for Systematic Treatment Decisions in Recurrent or Metastatic Head and Neck Squamous Cell Carcinoma: Perspectives from Experts and Junior Doctors

Running head: ChatGPT-assisted staging and treatment of HNSCC

Danfang Yan, Lihong Wang, Liming Huang , Kejia Cheng, Yu Huang, Yangyang Bao, Xin Yin, Mengye He, Huiyong Zhu, Senxiang Yan

Supplementary table

Patient characteristics

	Age	Gender	KPS	Tumor site	Previous treatment			CPS	Imaging results	Symptoms	Interval (months)
					Surgery	RT	CT				
Patient 1	70	Male	70	Oropharynx	Yes	Yes	No	10	Local recurrent	Dysphagia and pain	24
Patient 2	47	Male	90	Maxillary sinus	Yes	Yes	Yes	10	Local recurrent and metastatic	Pain	12
Patient 3	59	Male	90	Oropharynx	Yes	Yes	No	NA	Metastatic	None	3
Patient 4	46	Male	80	Oropharynx	Yes	Yes	Yes	60	Region recurrent	None	36
Patient 5	71	Male	90	Laryngeal	Yes	No	No	100	Local and region recurrent	Dysphagia and pain	34
Patient 6	72	Female	80	Oral	Yes	No	No	NA	Region recurrent	None	6
Patient 7	73	Female	70	Oral	Yes	No	No	<1	Local and region recurrent	Dysphagia and pain	7
Patient 8	59	Male	70	Oral	No	Yes	Yes	<1	Local recurrent	Pain	5
Patient 9	56	Male	60	Hypopharyngeal	Yes	Yes	Yes	NA	Metastatic	Pain	12
Patient 10	46	Male	70	Hypopharyngeal	Yes	Yes	Yes	60	Local recurrent and metastatic	Dysphagia and pain	12
Patient 11	60	Male	80	Oral	Yes	Yes	Yes	20	Local recurrent	Pain	9
Patient 12	76	Male	70	Maxillary sinus	Yes	Yes	No	NA	Local recurrent and metastatic	Pain	18

CPS: combined positive score; CT: chemotherapy KPS: Karnofsky performance status; RT: radiotherapy
